# Extreme temperature fluctuations in laboratory models of the mid-latitude atmospheric circulation

**DOI:** 10.1038/s41598-023-47724-2

**Published:** 2023-11-27

**Authors:** Miklós Vincze, Cathrine Hancock, Uwe Harlander, Costanza Rodda, Kevin Speer

**Affiliations:** 1 HUN-REN–ELTE Theoretical Physics Research Group, Budapest, 1117 Hungary; 2 Institute of Earth Physics and Space Science (HUN-REN EPSS), Sopron, 9400 Hungary; 3 Geophysical Fluid Dynamics Institute, Florida State University, Tallahassee, FL 32306-4360 USA; 4 Department of Aerodynamics and Fluid Mechanics, Brandenburg University of Technology Cottbus–Senftenberg, Cottbus, 03046 Germany; 5 Department of Civil and Environmental Engineering, Imperial College London, London, SW7 2AZ England, UK

**Keywords:** Fluid dynamics, Climate sciences, Atmospheric science, Climate change

## Abstract

Using two laboratory-scale conceptual fluid dynamic models of the mid-latitude atmospheric circulation we investigate the statistical properties of pointwise temperature signals obtained in long experiment runs. We explore how the average “equator-to-pole” temperature contrast influences the range and the jump distribution of extreme temperature fluctuations, the ratio of the frequencies of rapid cooling and warming events, and the persistence of “weather” in the set-ups. We find simple combinations of the control parameters—temperature gradient, rotation rate and geometric dimensions–which appear to determine certain scaling properties of these statistics, shedding light on the underlying dynamics of the Rossby wave-related elements of the mid-latitude weather variability.

## Introduction

The average temperature contrast between the subtropical and polar regions of Earth influences the frequency at which extreme (“abnormal”) temperatures occur at mid-latitudes. This connection has been subject to extensive research in the past decades, involving satellite and station data analysis^1^, simulations in global climate models^2,3^, and even experiments in conceptual laboratory settings. The observed changes in the persistence of certain weather situations, and their causal links to the weakening meridional temperature contrast also attract increasing attention in the climate science community. The average equator-to-pole temperature difference parameter $\langle \Delta T \rangle$ of the Northern Hemisphere lower troposphere exhibits a decreasing trend throughout the past decades^4,5^, as the Arctic has warmed nearly four times faster than the globe^6^ (“Arctic amplification”), and this climatic process affects the dynamics of atmospheric Rossby waves, or planetary waves, and the spatio-temporal distribution of weather patterns at mid-latitudes.

Large-scale Rossby waves are the dominant features of the Terrestrial weather system that form as a result of baroclinic instability in the atmosphere^7^. They help transfer warm air from the low latitudes to the poles and cold air backwards, attempting to restore equilibrium. The characteristic spatial scales—zonal wavelengths—of Rossby waves are known to depend on $\langle \Delta T \rangle$; generally, lower $\langle \Delta T \rangle$ yields smaller coherent structures. Furthermore, not only the length scales associated with these waves are influenced by $\langle \Delta T \rangle$, but also the speed at which they drift eastward: smaller meridional temperature contrast typically yields slower drift. Thus, the variability and persistence of temperature fluctuations at a given mid-latitude geographic location are affected both by the characteristic spatial scale of the meandering borderline around the cold polar regions and the speed of the pattern’s propagation.

The differentially heated rotating annulus is a widely studied experimental model in which the fundamental underlying dynamics of baroclinic instability, Rossby waves, and cyclogenesis can be reproduced to a conceptual level, obeying the principle of hydrodynamic similarity. The arrangement of this set-up has been introduced independently by the groups of David Fultz^8^ and Raymond Hide^9^ in the 1950s, and similar experiments have since been applied to, e.g., the investigation of Rossby wave dispersion and wave-wave interactions^10,11^, daily temperature statistics^12^, the spreading of passive tracers^13^, the excitation of inertial gravity waves^14,15^ in the atmosphere, and even for the validation and fine-tuning of numerical models and methods for weather forecasting^16,17^.

Further experimental work conducted in baroclinic rotating annulus setups addressed the issue of temperature fluctuations in an ensemble of experiments with a continuously changing temperature contrast^18^, an essential aspect of the ongoing climate change. The basic relationships between the occurrence of extremely low or high temperature values and the meridional temperature contrast parameter $\Delta T$ have also been investigated in our more recent experiments^19,20^, of which the present work is a natural continuation and extension.

Here we do not aspire to provide a detailed description of the flow features in the studied setting (as it has been done in the aforementioned previous research), instead we limit our scope to the scaling properties of various statistical measures characterising the temperature fluctuations in this atmosphere-like sideways-convective rotating configuration. In order to explore a wide range of the relevant parameters, we contrast results from two series of experiments in set-ups with markedly different geometrical dimensions. We put a special emphasis on the analysis of “extremes” in temperatures and temperature jumps. In the investigated quasi-stationary signals we consider a value “extreme” when it falls out of the range containing 90% of all measured fluctuations around the long-time average (following appropriate detrending). We also evaluate the shape and scale parameters of the extreme value distributions of the temperature records, and their scaling with the control parametrs. The probability density function (PDF), and hence, the extreme value statistics of the temperature fluctuation time series in complex systems - including Earth’s climate system - are not independent from the spectral properties of the signal and vice versa. (Note, that this is far from being a trivial statement: generally, a randomly shuffled temperature time series would still have the same PDF as the original, but all spectral information would be totally destroyed.)

The seminal paradigm-changing minimal model of Hasselmann^21^ successfully explained the “red noise” character of climate variability based on the principle that one can treat the slowly changing component of the variability as a deterministic linear system, and the high-frequency effects as an uncorrelated additive noise. However, it can be shown that such additive noise models cannot produce the non-Gaussian PDFs of temperature fluctuations often observed in nature^22^, and therefore fail to account for the scaling of the extreme tails of the PDFs.

Models applying correlated additive and multiplicative (CAM) noise forcing, however, can produce “heavy”- (power-law-) tailed distributions while also maintaining the red noise behavior of the spectra. The frequency spectrum $F(\omega )$ has the form $F(\omega )\propto 1/(\lambda ^2 + \omega ^2)$, where parameter $\lambda$ is associated with the critical frequency, below which the spectrum is white noise-like, and above which it follows a power law. This spectrum shape is identical to the case of purely additive noise, with the important difference that here $\lambda$ also depends on the multiplicative noise^23^. As for the PDF, the power law exponent $\alpha$ of the tail ($p(x) \propto |x|^{-\alpha }$) is also a function of the coupling constant of the multiplicative noise. In the CAM model $\alpha$ is found to follow an increasing linear relation with $\lambda$, which means that weaker damping (i.e. smaller cut-off frequency $\lambda$) results in heavier tails (smaller $\alpha$), i.e. larger extreme values are expected.

To explore whether the dynamics of temperature fluctuations in the laboratory experiments behaves similarly to a CAM model, we investigate how certain spectral properties (cut-off frequencies, and characteristic timescales) of the recorded temperature signals scale with the thermal forcing, and discuss whether their change is consistent with the scaling of the ranges of extreme temperatures. Our results may hopefully be applicable for a better understanding of the relevant scale parameters in the actual mid-latitude weather variability.

## Set-up and data acquisition

**Figure 1:**
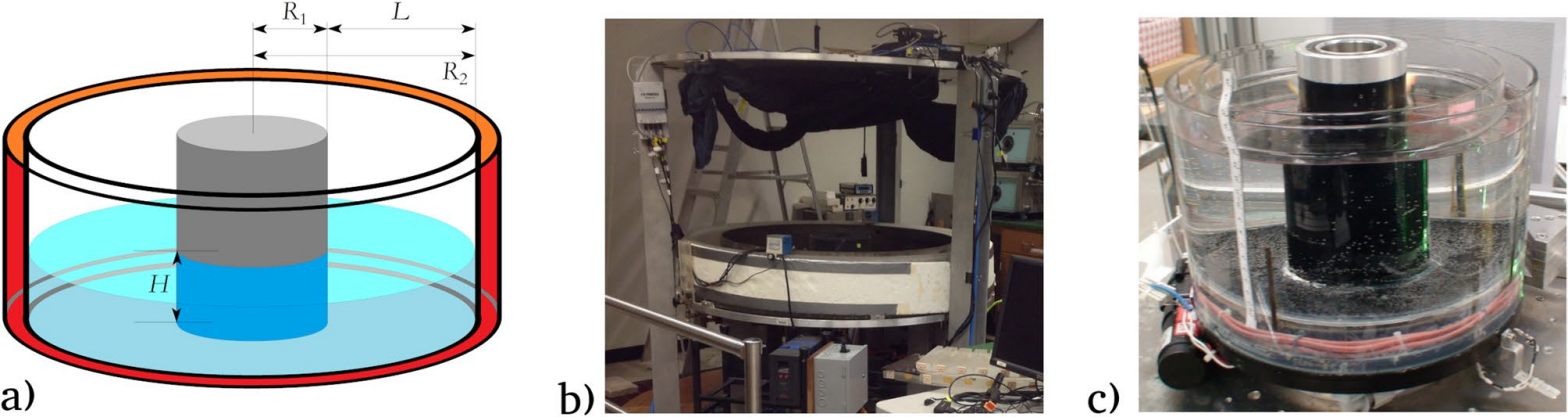
The experimental setups. **(a)** Sketch of the rotating annulus configuration and its most relevant dimensions (cf. Table 1). **(b)** A photo of the GFDI large annulus setup. **(c)** A photo of the BTU small annulus setup. (In this picture water level *H* does not match the one used in the present experiments.) The figure in panel (a) was created using KolourPaint for Ubuntu linux, Release 20.12.3 (freely available and downloadable at https://apps.kde.org/kolourpaint/).

The basic layout of the rotating annulus configuration is sketched in Fig. 1a. The tank is mounted on a turntable revolving around its axis of symmetry at angular velocity $\Omega$ in counterclockwise direction ($\Omega > 0$) and is divided into three sections by heat conductive coaxial cylindrical walls. The innermost domain of radius $R_1$ is referred to as the “cold bath”, where water of constant temperature $T_1$ (below room temperature) is circulated through a cooling thermostat (chiller). A separate regulated closed loop water circuit keeps the “warm bath”, i. e. the outermost annular gap, and its sidewall of radius $R_2$ at a higher prescribed temperature $T_2 (> T_1)$. The inner annular cavity of gap width $L = R_2 - R_1$ forms the experimental domain, and is filled up with the working fluid—water—up to height level *H*. The fluid surface is free, and the flow is driven by the buoyancy flux maintained by the temperature contrast $\Delta T = T_2 - T_1$ between the cylindrical walls. This configuration is a barebone representation of the atmosphere at the mid-latitudes of the Northern Hemisphere with the inner “cold” cylinder modeling the cold polar regions and the “warm” outer rim the subtropics.

Our experiments were conducted in two set-ups with different dimensions summarized in Table 1. The “large annulus” (Fig. 1b) is located in the Geophysical Fluid Dynamics Institute (GFDI) of the Florida State University, while the “small annulus” (Fig. 1c) belongs to the Department of Aerodynamics and Fluid Mechanics of the Brandenburg University of Technology (BTU); hereafter, the two respective series of experiments will thus be referred to as GFDI and BTU runs.

**Table 1:** The parameters of the two experiment configurations.

parameter	GFDI (large annulus)	BTU (small annulus)
gap inner radius $R_1$ [m]	0.16	0.045
gap outer radius $R_2$ [m]	0.61	0.125
gapwidth *L* [m]	0.45	0.075
water level *H* [m]	0.08	0.05
thermometer level *h* [m]	0.01	0.006
angular velocity $\Omega$ [rad/s]	1.047	2.096
Taylor number *Ta*	$1.01\cdot 10^{12}$	$8.32\cdot 10^{8}$
thermal Rossby number $Ro_T$	0.001–0.0029	0.0032–0.011
mean temperature contrast $\langle \Delta T \rangle$ [$^\circ$C]	5.5–15.9	2.9–10.9
number of runs	12	7

This work focused on the analysis of temperature time series obtained from fixed co-rotating thermocouples in the two annulus setups. In the case of the GFDI apparatus, standard K-type thermocouples with a measurement precision of $\delta T \approx 0.001^{\circ }$C were utilized, whereas, the BTU setup used Ahlborn ALMEMO NiCr sensors with $\delta T \approx 0.05^{\circ }$C. The temperature records were acquired at 1 Hz sample rate in both configurations by co-rotating data loggers mounted on the turntables. The thermometer placed in the working fluid was fixed at the mid-radius of the gap, i.e. at distance $R = (R_1 + R_2)/2$ from the axis of symmetry and at a small height of *h* from the bottom of the tank (see Table 1). It is to be noted that in both settings *h* is already above the Ekman boundary layer^7^, whose characteristic scale is determined by $\Omega$ and the kinematic viscosity of the working fluid. Identical thermometers were installed in the cold and warm baths to record the time development of the thermal boundary conditions.

The experiments were performed as follows. After filling up the tank with water, first the heating and cooling systems were turned on and then rotation was initiated. In a transient phase of ca. 4000 s after rotation was started the temperature contrast $\Delta T$ exhibited a steep exponential saturation, followed by a small-slope plateau. Our analysis was limited to the latter quasi-stationary phase. The length of the evaluated temperature signals was 16 000 s (i.e. around 2666 revolutions, or “days”) in the case of the GFDI runs, and 25 000 s (i.e. around 8333 revolutions, or “days”) for the BTU runs.

## Results

### The range of temperature fluctuations

We evaluated the statistical properties of temperature fluctuations *T*(*t*) over time *t*, acquired by the sensor in the experimental domain throughout the aforementioned quasi-stationary part of the runs. The time-average of the temperature contrast $\langle \Delta T (t) \rangle \equiv \langle T_2(t) - T_1(t) \rangle$ served as an adjustable control parameter, whereas rotation rate $\Omega$ and water depth *H* were fixed in both series of experiments.

The Taylor number *Ta*, a nondimensional parameter for quantifying the effect of the Coriolis acceleration relative to that of the viscous drag reads as

1 $$\begin{aligned} Ta=\frac{4\Omega ^2 L^5}{\nu ^2 H}, \end{aligned}$$

where $\nu = 1.004 \times 10^{-6}$ m$^2$/s is the kinematic viscosity of the fluid (water). Since $\Omega$, *H* and the gapwidth *L* did not vary within the two series of experiments, *Ta* was also fixed in both configurations, yielding $Ta_{\textrm{GFDI}} = 1.01\cdot 10^{12}$ and $Ta_{\textrm{BTU}} = 8.32\cdot 10^{8}$ for the GFDI and BTU experiments, respectively.

The other important nondimensional parameter of thermally driven flows subject to Coriolis force is the thermal Rossby number $Ro_T$ (also known as Hide number) defined as

2 $$\begin{aligned} {\text{ Ro }}_T=\frac{\alpha g H \langle \Delta T \rangle }{4 \Omega ^2 L^2}\hspace{5.0pt}, \end{aligned}$$

where $g = 9.81$ m/s$^2$ is the acceleration of gravity, and $\alpha = 2.07\times 10^{-4}$ 1/K represents the volumetric thermal expansion coefficient of water around room temperature. (The typical scale of $Ro_T$ for Earth’s Rossby waves is $\mathcal {O}(Ro_T) = 10^{-2}$.) Note, that although the $\langle \Delta T \rangle$ intervals of the GFDI and BTU series overlap (see Table 1), they cover separate domains when expressed using $Ro_T$ as a nondimensional temperature scale.

**Figure 2:**
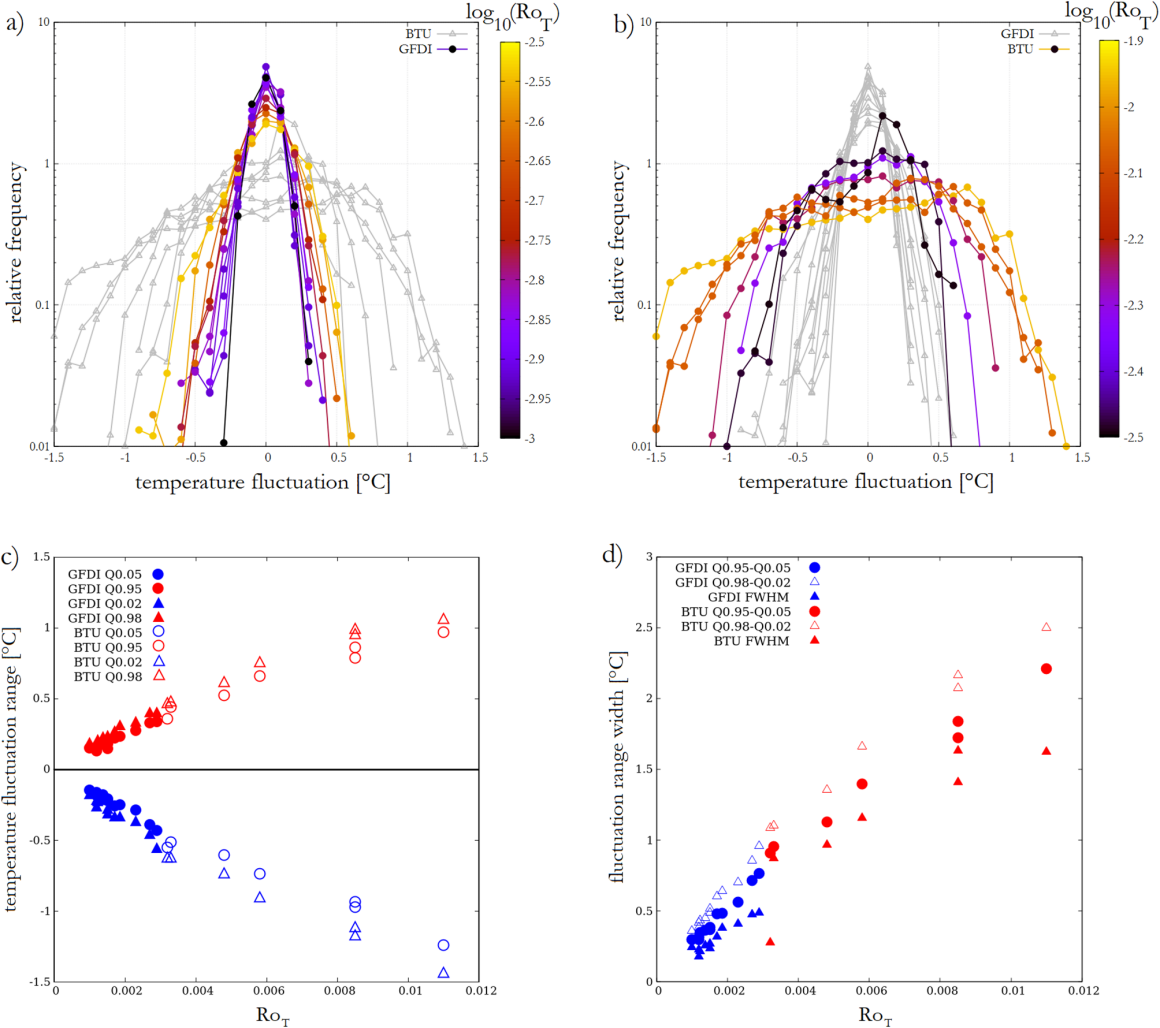
The scaling of temperature fluctuations. **(a)** and **(b)** Histograms of temperature fluctuation signals *T*(*t*) acquired in the vicinity of the bottom close to the mid-radius of the annular gap. The data points are repeated in both panels but those from the GFDI and BTU runs are highlighted by coloring—following $\log _{10} Ro_T$—at panels **a** and **b**, respectively. **(c)** Limit points of the intervals associated with different measures of the range of detrended temperature signals *T*(*t*) based on the percentiles indicated in the legend, as a function of thermal Rossby number $Ro_T$. Filled blue and red data points correspond to the lower and higher endpoints of the intervals, respectively, for the GFDI runs, unfilled symbols represent the analogous endpoints for the BTU experiments. **(d)** The lengths of the intervals covering the range in which 90% (circles) and 96% (empty triangles) of the values of *T*(*t*) scatter around zero, and the full width at half maximum (FWHM, filled triangles) of the corresponding distribution. The coloring indicates the series of experiment runs (GFDI—blue, BTU—red). The figure was created using Gnuplot version 5.2, Release 5.2.8 (freely available and downloadable at http://www.gnuplot.info/).

The analyzed time series *T*(*t*) were obtained following the removal of a 7th-order polynomial trend from the raw signals, thus $\langle T(t) \rangle = 0$ holds. The histograms of *T*(*t*) from all experiments are shown in Fig. 2a and b. The data points are identical in both panels, with GFDI and BTU measurements highlighted in panels a) and b), respectively. In each panel the data points are colored with respect to the (logarithm of the) $Ro_T$ of each run, and analogously for the BTU runs in panel (b). As visible, the runs with higher $\langle \Delta T \rangle$ (and hence higher $Ro_T$) exhibited wider range of temperature fluctuations for both tank configurations. Note, that the vertical scale in panels (a) and (b) is logarithmic, therefore a histogram following a normal distribution would be parabolic here. The “extreme” range in which the measured temperature signal tended to fluctuate around zero was expressed using the quantiles of these histograms, corresponding to the 5th and 95th percentiles (marked as Q0.05 and Q0.95, respectively) of the empirical distribution, as well as for the 2nd and 98th percentiles (Q0.02 and Q0.98).

We found that a remarkably good collapse of data across the GFDI and BTU results is achieved if these (dimensional) temperature fluctuation ranges are plotted against $Ro_T$. Figure 2c shows the aforementioned Q0.02, Q0.05, Q0.95, Q0.98 quantiles as a function of $Ro_T$ for the GFDI (filled red and blue symbols, see legend) and the BTU (unfilled red and blue symbols) runs. The characteristic ranges of their temperature fluctuation distributions seem to follow the same, roughly linear scaling, strongly implying that—within the studied flow regimes—$Ro_T$ provides an appropriate parameter to describe the extreme excursions of temperature in rotating annulus experiments.

Figure 2d presents the width of the extreme ranges, i.e. the Q0.05–Q0.95 (circles) and the Q0.02–Q0.98 (empty triangles) intervals for both series (GFDI blue, BTU red). To quantify the more typical fluctuations the full width at half maximum (FWHM) of the distribution is also plotted for each run (filled triangles). (FWHM is a commonly used standard parameter for measuring the width of distribution, spectral peak, or any bump-like pulse, that is easy to obtain and does not require any *a priori* knowledge about the function. In the case of a Gaussian distribution FWHM is directly proprtional to the standard deviation $\sigma$: $\textrm{FWHM}=2\sqrt{2\ln 2}\sigma \approx 2.335 \sigma$.) These measures of width also appear to scale with $Ro_T$ in a fairly consistent manner.

It is to be noted that this collapse of data also implies that although generally $Ro_T$ is not the only nondimensional parameter to be considered for the dynamics of Rossby waves, in the studied domain of the $Ta-Ro_T$ parameter plane the range of temperature fluctuations does not appear to depend on Taylor number *Ta*. (The *Ta*-values for the two configurations were of different orders of magnitude, see Table 1).

**Figure 3:**
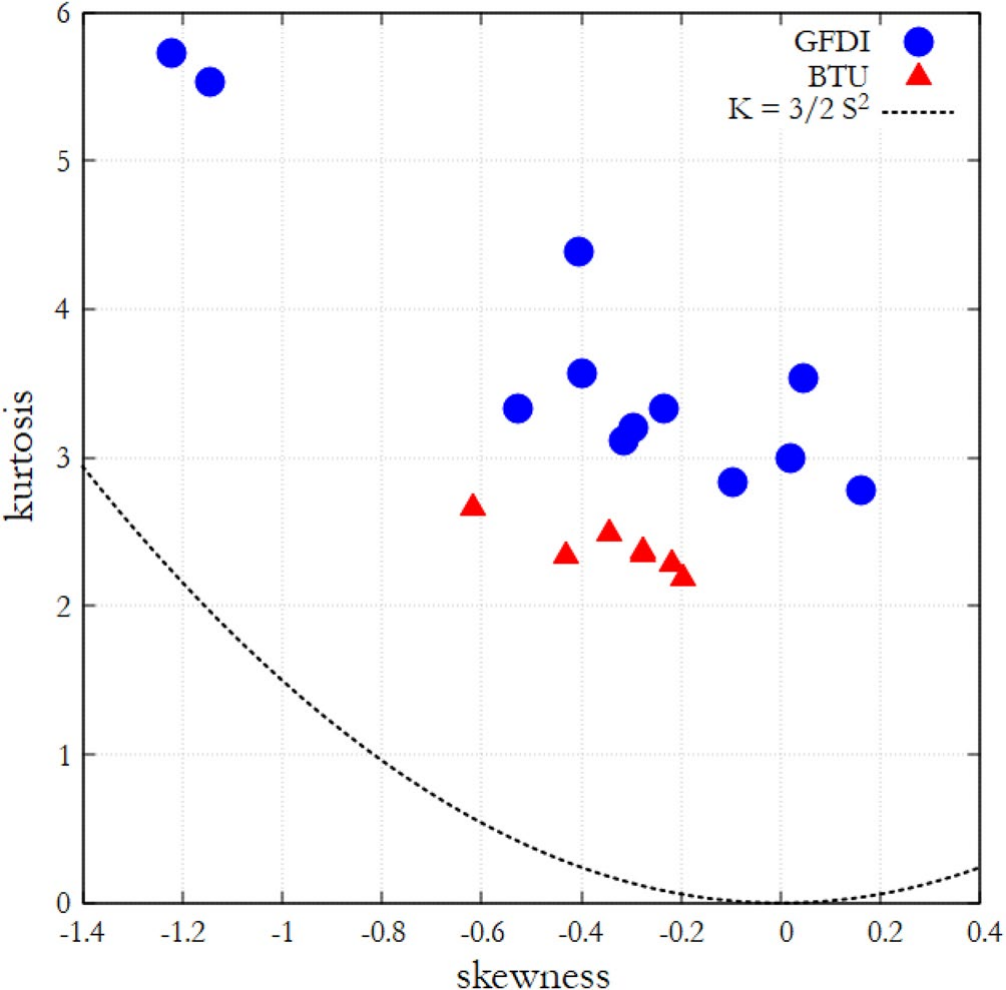
Kurtosis *K* versus skewness *S* of the temperature signals. The coloring indicates the series of experiment runs (GFDI—blue, BTU—red) and the dashed line denotes the function $K=(3/2) S^2$. The figure was created using Gnuplot version 5.2, Release 5.2.8 (freely available and downloadable at http://www.gnuplot.info/).

As visible from the histograms of Fig. 2a and b, the distributions of the temperature fluctuation signals are dominantly left skewed and have rather fat tails. The values of kurtosis *K* of the samples are plotted against their skewness *S* in Fig. 3 for both series of experiments. The implications of the fact that all data points scatter above the $K=(3/2) S^2$ (dashed) curve will be discussed later, in the section “Discussion and conclusions”. It is also to be noted that no obvious correlation could be established between *S* or *K*, and the control parameters $Ro_T$ or $\langle \Delta T \rangle$.

### The scale and shape parameters of the extreme tails

A common approach to explore the probabilities of events associated with extremely high temperatures in the climate system is fitting the Generalized Pareto Distribution (GPD)—a widely applicable model for the tails of other distributions—to the empirical probability of temperature *T*(*t*) exceeding a value $x > 0$ among those data points that are higher than a threshold $u > 0$^24,25^. This conditional probability *p* is given by

3 $$\begin{aligned} p(T(t)-u>x \,\,|\,\, T(t) > u)=\Big ({1+\frac{\xi x}{\sigma _u}}\Big )^{-\frac{1}{\xi }}, \end{aligned}$$

where $\sigma _u > 0$ and $\xi \ne 0$ denote the so-called scale and shape parameters of the distribution, respectively. The problem of selecting the lowest threshold $u_0$ above which an empirical distribution can be considered to be a member of the GPD family is not trivial. However, it can be easily shown that once the distribution of the excesses of $u_0$ exhibits GPD-like character, this remains valid for all thresholds $u>u_0$, too. Then, theoretically, the shape parameter $\xi$ is independent from the selected *u*, whereas $\sigma _u$ scales linearly with *u* in such a manner that the re-parameterized $\sigma ^*=\sigma _u-\xi u$ should also remain constant for all thresholds $u\ge u_0$.

One can estimate $\xi$ and $\sigma ^*$ using the multiple threshold method^24,26^, where the log-likelihood function^24^ $\mathcal {L}(\xi _u,\sigma _u)$ corresponding to (3) is maximized numerically for threshold excesses $x-u > 0$, and the process is repeated for a wider range of thresholds *u*. The estimates for $\xi$ and $\sigma ^*$ can then be obtained by fitting the linear function $\sigma _u(u)=\xi u + \sigma ^*$ to the obtained maximum likelihood estimates of the scale parameter versus *u*. Plotting the function $\sigma _u(u)$ is also useful for finding the lowest threshold $u_0$ above which the function can be considered linear, indicating the validity of the GPD approximation. In all of our experiments $u_0$ fell above the Q0.95 quantile of temperature fluctuations.

**Figure 4:**
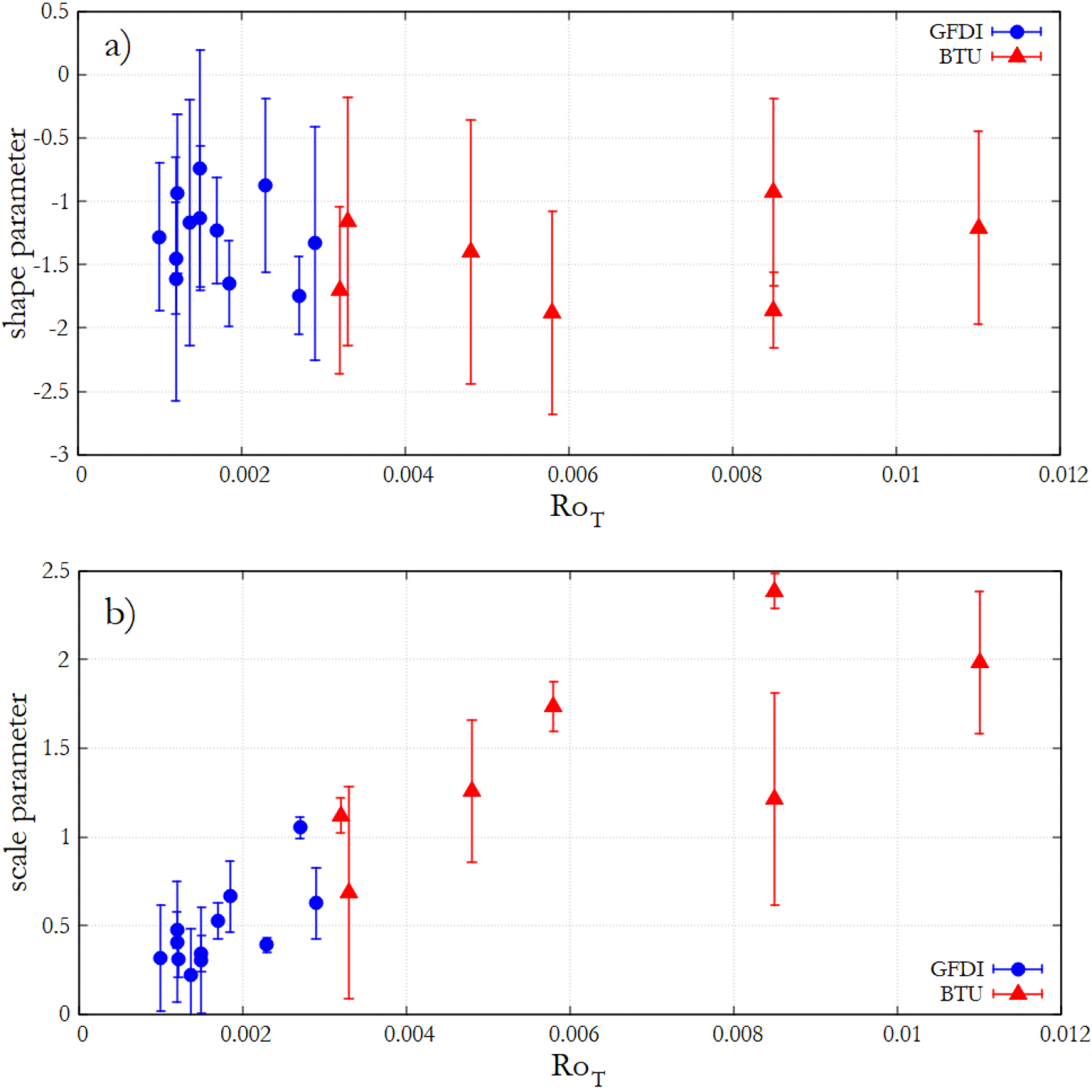
Shape and scale parameters of the temperature fluctuations. Shape parameter $\xi$ **(a)**, and re-parameterized scale parameter $\sigma ^*$ **(b)** of the Generalized Pareto Distributions fitted to the extreme fluctuations of the temperature time series as a function of thermal Rossby number $Ro_T$. The coloring indicates the series of experiment runs (GFDI—blue, BTU—red). The error bars represent the root mean square error of the linear fits of the $\sigma _u(u)$ function obtained from the maximum likelihood estimates with multiple thresholds *u*. The figure was created using Gnuplot version 5.2, Release 5.2.8 (freely available and downloadable at http://www.gnuplot.info/).

The resulting estimates of these parameters are presented in Fig. 4 for both series of experiments as a function of $Ro_T$. As visible in panel a), data points for the shape parameter $\xi$ scatter around the mean value $\xi =-1.33$ (with a standard deviation of 0.41) without any obvious trend. The scale parameter $\sigma ^*$, however, clearly appears to increase linearly with $Ro_T$, and the data from the two experiment configurations, again, exhibit a rather good collapse of data.

These findings indicate that the exponent of the extreme power-law tail of the temperature distribution does not depend on the “meridional” temperature contrast of the model, and therefore, in accordance with the widening of the histograms—already observed in Fig. 2 in terms of the quantiles—the highest extreme temperature values which may be observed in the system (scaling with $\sigma ^*$) also increase linearly with the thermal Rossby number $Ro_T$.

### The range of temperature “jumps”

Next, we investigated the extremes in the rate of temperature change over time, and their scaling properties with respect to the parameters of the experiments. In the raw time series the finite temperature resolution $\delta T$ of the thermometers and the frequent sampling (relative to the characteristic timescales in the flow) unavoidably produce time intervals of seemingly constant temperature and step-wise changes of magnitude $\delta T$ in both directions. Thus, calculating a pure forward difference of the raw signals would yield a time series consisting of the values 0 and $\pm \delta T$ only (or, with a lower sampling rate, its integer multiples). Hence, for this analysis we calculated a running mean of *T*(*t*) using a window of 60 s—i.e. 60 data points, as the sample rate was 1 Hz, translating to 10 and 20 revolutions in the GFDI and BTU set-ups, respectively—and then the forward difference of this smoothed signal. Analogously to the previously discussed case of the temperature values themselves, we considered a temporal change of temperature “extreme” if it fell out of the Q0.05–Q0.95 percentile range of the respective distribution of (smoothed) temporal temperature changes, hereafter referred to as “jumps”. Our analysis focused on the values of these percentiles.

As reported by Gyüre et al.^12^, daily temperature changes in weather station records exhibit a robust asymmetry: increasing steps in the time series occur more frequently and have lower average magnitude than decreasing steps, implying that the cooling caused by cold fronts penetrating a region close to the surface is typically more rapid than the temperature increase due to warm fronts. Gyüre et al. also demonstrated this feature in a rotating annulus experiment.

**Figure 5:**
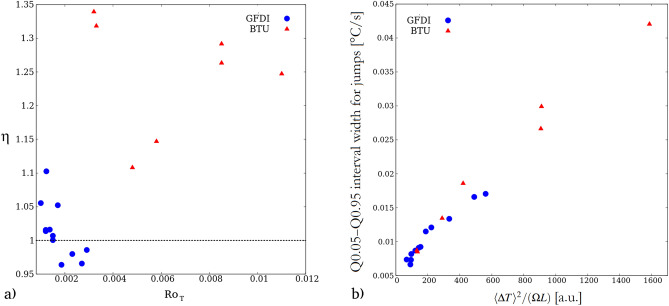
The scaling of temperature jumps. **(a)** Extreme skewness parameter $\eta$ of the distribution of temperature jumps as a function of thermal Rossby number $Ro_T$ for the two series of experiments (see legend). **(b)** The length of the interval in which 90% of the jump distribution scatter around zero (i.e., the Q0.05–Q0.95 interval) for the two series of experiments (see legend) plotted against the parameter combination proportional to the characteristic temperature jump scale associated with an advection dominated heat transfer (cf. Eq. 4). The figure was created using Gnuplot version 5.2, Release 5.2.8 (freely available and downloadable at http://www.gnuplot.info/).

When considering the extreme changes in temperature, the same asymmetry can be tested by introducing an “extreme skewness parameter” $\eta = |Q0.05|/|Q0.95|$, i.e. the ratio of the absolute values of the 5th and 95th percentiles (the mean of the distribution is zero since *T*(*t*) is stationary). This parameter was constructed in order to get a robust measure that is sensitive to the asymmetry of the extreme tails of the distribution, which is not necessarily captured by the sample skewness. $\eta$ is shown as a function of thermal Rossby number $Ro_T$ in Fig. 5a for both series of experiments (GFDI with blue circles, BTU with red triangles). Although no clear trend can be established, it is apparent that the vast majority of the data points scatter above the $\eta = 1$ line, implying that extremely rapid drops of temperature are significantly more common than abrupt warming jumps.

Unlike the magnitude of extreme temperature fluctuations the width of the Q0.05–Q0.95 interval of the jump distribution is not expected to scale with $Ro_T$. The time derivative of a temperature signal obviously depends on the characteristic timescale of the process, whereas $Ro_T$ is timescale-independent by definition. Indeed, one way to introduce $Ro_T$ is to treat it as the squared ratio of two characteristic temporal units: the time it takes for the flow to cross the horizontal domain of size *L* and the period of rotation (proportional to $1/\Omega$)^7^.

A further timescale of interest could be the one associated with conductive heat transfer. However, taking the molecular thermal diffusivity of water $\kappa \sim 10^{-7}$ m$^2$/s and either the horizontal or the vertical length scale of the system *L* (or *H*) one finds that the conductive time scale can be estimated as $L^2/\kappa$ (or $H^2/\kappa$), yielding a range of $\sim 10^4$–$10^6$ s, much larger than the typical scales on which the “weather” fluctuates in both experimental set-ups.

Therefore, one can assume that the change of temperature (and hence, density) of any given incompressible water parcel in the flow is negligible, i.e.

4 $$\begin{aligned} \frac{dT}{dt} \equiv \frac{\partial T}{\partial t} + \vec {u} \nabla T \approx 0 \end{aligned}$$

holds, where $d \cdot /dt$ represents the total (Lagrangian) time derivative along the trajectory of a parcel, $\partial \cdot / \partial t$ marks the change of temperature measured at a given fixed measurement location $\vec {r}$, and $\vec {u}(\vec {r},t)$ denotes the velocity of the flow at $\vec {r}$.

With the assumption of (4) the measured time derivatives of the temperature signals at a fixed measurement location are determined by the $\vec {u} \nabla T$ term, whose scaling properties can be obtained by estimating the characteristic velocity scale of the flow *U* and expressing the typical temperature gradient as $\nabla T \sim \langle \Delta T \rangle /H$. For the latter estimate we used *H* since the vertical temperature gradient in the system is larger than the horizontal as both scale with $\langle \Delta T \rangle$, but $H<L$ in both experimental tanks.

The aforementioned characteristic horizontal velocity *U* of a buoyancy-driven flow in (quasi-) geostrophic equilibrium—where the pressure gradient force caused by the temperature differences are balanced by the Coriolis force—can be estimated as^27^

5 $$\begin{aligned} U\approx \frac{\alpha g H \langle \Delta T \rangle }{2\Omega L}. \end{aligned}$$

Thus, the product $\vec {u} \nabla T$ is expected to depend on the combination $U \langle \Delta T \rangle /H$, yielding $\alpha g \langle \Delta T \rangle ^2 / (2\Omega L)$, that is proportional to $\langle \Delta T \rangle ^2 /(\Omega L)$, since the values of $\alpha$ and *g* are the same for both configurations.

The widths of the Q0.05–Q0.95 intervals for the jump distributions of all runs are plotted against $\langle \Delta T \rangle ^2 /(\Omega L)$ in Fig. 5b. The data points from the GFDI (blue) and BTU (red) runs exhibit a fairly good data collapse, suggesting that the extreme fluctuations of jumps indeed tend to depend on the time scale associated with advective heat transfer.

### Power spectra and the scaling of persistence

**Figure 6:**
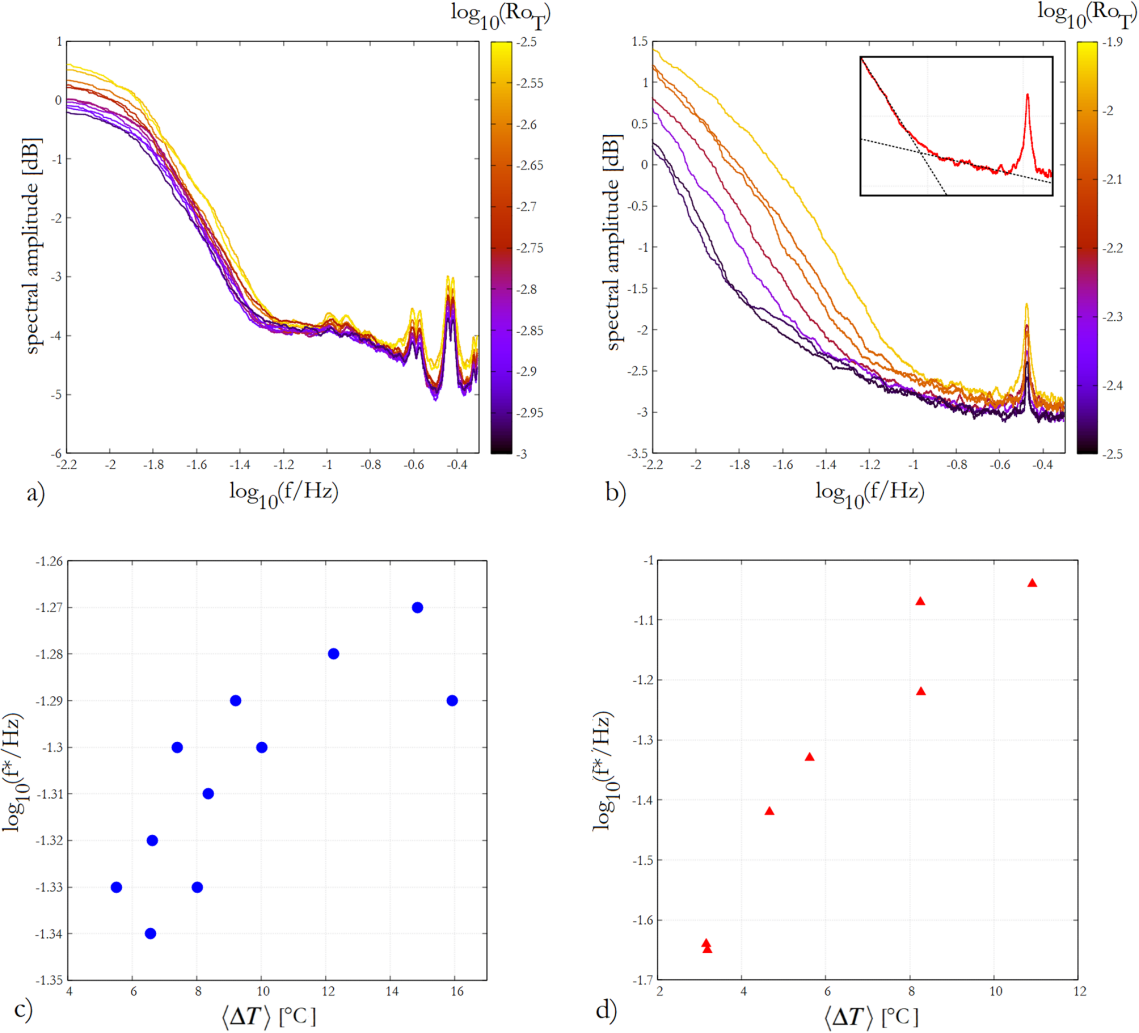
Fourier spectra of temperature signals. Double logarithmic Fourier spectra of the detrended temperature signals *T*(*t*) from the GFDI **(a)** and BTU runs **(b)**. The spectra were obtained using the Lomb–Scargle Fourier transform method using the REDFIT package^28^, and were smoothed with a 201-point running averaging. The color scale represents the logarithm of the thermal Rossby number (i.e. the nondimensional temperature difference scale). Panels **(c)** and **(d)** show the logarithm of the threshold frequency $f^*$—obtained as the frequency where the power law fits for the tail and the mid-frequency band of the spectra intersect, as shown in the inset of panel **(b)**—as a function of the temperature contrast $\langle \Delta T\rangle$ for the GFDI and BTU runs, respectively. The figure was created using Gnuplot version 5.2, Release 5.2.8 (freely available and downloadable at http://www.gnuplot.info/).

We addressed the spectral features of the *T*(*t*) signals and explored the $\langle \Delta T \rangle$-dependence of two relevant timescales (or, equivalently, frequency scales): the threshold frequency $f^*$ above which the fluctuations resemble an uncorrelated noise, and the one associated with the spectral domain, in which the persistence of the “weather” is the highest.

The Fourier spectra of *T*(*t*) from all experiment runs—obtained using the Lomb–Scargle Fourier method and the REDFIT software package^28^, then smoothed with a 201-point running averaging in the frequency domain for better visibility—are shown in Fig. 6a and b for the GFDI, and BTU set-ups, respectively. The coloring represents the (logarithm of) $Ro_T$ that is proportional to $\langle \Delta T \rangle$ within both experiment configurations. The sharp high frequency peak, and its harmonics correspond to the rotation rate of the tank, which were identical in all runs of the given apparatus (cf. Table 1). Clearly, each spectrum exhibits a red noise character up until a certain threshold frequency $f^*$ around which a distinct kink is found. $f^*$ serves as a measure for the separation between the “more correlated” and “less uncorrelated” fluctuation timescales, and a clear increasing trend of its value with the temperature contrast parameter can be observed in both series of experiments, as shown also in Fig. 6c and d for the GFDI and BTU runs, respectively. The small inset in panel b) provides an exemplary illustration of the procedure we used to approximate $f^*$ by finding the intersection point of linear fits to the high-frequency range (but below aforementioned peaks) and the mid-frequency domain of the doubly logarithmic-scaled spectra.

Next, we explored the “predictability” of time series *T*(*t*) in terms of persistence *P*. In meteorology, persistence refers to the extent at which a given day’s (or other time interval’s) weather can be used as a credible forecast for the following day(s). Persistence $P=0$ indicates an uncorrelated signal, while $P\approx 1$ implies a tendency of long intervals with similar values^29^. The persistence of a time series is closely related to its correlation properties. The autocorrelation function $A(\tau )$ of a time series *X*(*t*) of long-term memory shows a power-law decay $A(\tau ) = \langle X(t) X(t+\tau ) \rangle \sim \tau ^{-\alpha }$ with correlation exponent $0<\alpha <1$. The scaling properties of variance within intervals of length *s* can also be expressed in terms of the Hurst exponent *H* as $s^{2H}$. Persistence *P*, Hurst exponent^30^ *H*, and the correlation exponent $\alpha$ are connected by the following relationships: $P = 2H - 1$; $H = 1 - \alpha /2$. A Brownian noise yields $H = 0.5$, thus a $H>0.5$ scaling indicates the presence of memory in the system.

A convenient method to obtain an estimate of *H* is provided by the detrended fluctuation analysis (DFA) which measures the average magnitude of fluctuations as a function of the interval length *s* of the time series^31,32^. DFA has the adventage of being computationally robust and that it removes polynomial trends over each considered interval. Here we apply the DFA2 routine, where the number 2 refers to a quadratic polynomial used in the detrending.

**Figure 7:**
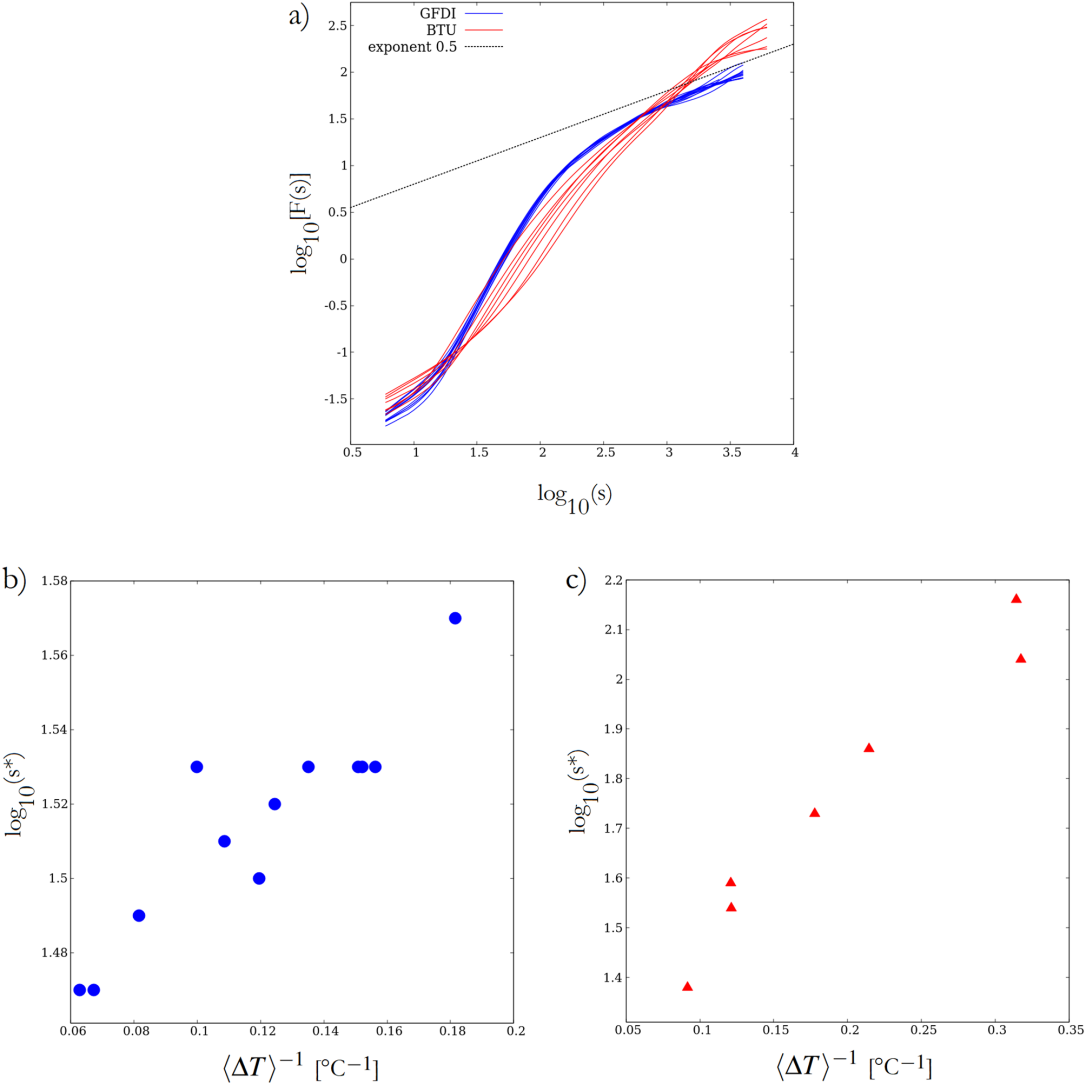
The persistence of temperature signals. **(a)** Double logarithmic DFA2 spectra of the detrended temperature signals *T*(*t*) from the two series of experiments (see legend). The dotted line represents the slope (scaling exponent) of 0.5 associated with an uncorrelated Brownian noise-type signal. The DFA2 spectra were generated using the code by J. Mietus et al. dfa.c available at: https://www.physionet.org/content/dfa/1.0.0/dfa.c. Panels **(b)** and **(c)** show the logarithm of the timescale associated with the steepest point of the double logarithmic DFA2 spectra, i.e. the largest local scaling exponent of the fluctuation ranges for the GFDI and BTU runs, respectively. The figure was created using Gnuplot version 5.2, Release 5.2.8 (freely available and downloadable at http://www.gnuplot.info/).

The DFA2 spectra of the *T*(*t*) signals are shown in Fig. 7a for the GFDI and BTU runs, with blue and red curves, respectively. The black solid line represents a slope of 0.5, associated with a Brownian process. (Note the double logarithmic scale.) To evaluate how the control parameter $\langle \Delta T \rangle$ affects the “weather in the tank”, we determined the timescale $s^*$ at which the largest local exponent *H* is found (i.e. where the slope of the log–log DFA2 spectrum is the steepest). Panels Fig. 7b and c show $s^*$ as a function of $\langle \Delta T \rangle ^{-1}$ for the GFDI and BTU experiments, respectively. Apparently, in both series of runs, the relationship is linear, indicating a roughly inverse proportionality between the logarithm of the “most persistent” timescale and the temperature contrast. In other words, the time scale at which the coarse-grained signal of temperature fluctuations is the most predictable appears to be larger for a smaller “meridional” temperature gradient. This observation also implies that for timescales below $s^*$ the predictability generally decreases with $\langle \Delta T \rangle$.

We note, that for the spectral measures $f^*$ and $s^*$ we were not able to find a combination of parameters that would yield a fairly good collapse of data from the two series of experiments like in the case of temperature fluctuations (Fig. 2) or in the case of jump distributions (Fig. 5).

## Discussion and conclusions

In the present work we reported on the analysis of temperature signals acquired from minimal laboratory models of Rossby waves in the mid-latitude atmosphere. We found that the range of temperature fluctuations and their extreme values scale with the thermal Rossby number $Ro_T$, a nondimensional parameter quantifying the ratio of the two most relevant hydrodynamic timescales in a thermally driven rotating flow. As the shape of the histograms (Fig. 2), the analysis of their skewness and kurtosis (Fig. 3) and the fitted scale and shape parameters (Fig. 4) of the extreme tails clearly reveal, these distributions are not Guassian (the Kolmogorov–Smirnov tests for normality we performed also reject the null hypothesis of normality with confidence limits of $\alpha > 0.99$ for all experiments).

As stated in the introduction, following the reasoning of Sura^22^, the non-normality of the PDFs indicates that the temperature fluctuations in our laboratory systems cannot be described statistically by the simplest Hasselmann-type quantitative model of climate variability, in which the “slow” changes are driven by a “fast” additive noise. However, we found that not only are the distributions becoming with power-law-like tails, but the low-frequency amplitudes of the (typical red noise-like) temperature fluctuation spectra also increase (and so does the characteristic frequency $f^*$) as the thermal forcing $\langle \Delta T \rangle$ increases. This behavior is qualitatively consistent with a simple one dimensional stochastic model that is able to produce non-Gaussian variability and red noise-like spectrum simultaneously, and where higher critical frequencies in the spectra correspond to heavier tails in the PDFs. Such a correlated additive and multiplicative (CAM) model for a variable *x* is described by the Stratonovich stochastic differential equation, which reads as

6 $$\begin{aligned} \frac{dx}{dt}=-\lambda x + (\phi x + 1)\eta + \eta ' - \phi \langle x \eta \rangle , \end{aligned}$$

where $\lambda$ denotes the “effective drift”, $\phi$ is a constant, and $\eta$ and $\eta '$ represent independent Gaussian white noise forcings. The second term of the right-hand side is the CAM term itself, in which the state-dependent $\phi x$ is multiplied by $\eta$, which (besides $\eta '$) also contributes to the additive (state-independent) noise, hence the two contributions of $\eta$ are correlated (positively, if $\phi > 0$, negatively if $\phi < 0$). Since the Stratonovich interpretation of stochastic differential equations is applied here, where the uncorrelated noise process is approximated with a continuous “Brownian motion” of a very short but still finite correlation time, the last term, which incorporates the correlation of the noise forcing $\eta$ with the state variable *x* is not exactly zero.

It has been demonstrated^22^ that the autocorrelation function $\rho (t)$ of *x*(*t*) governed by (6) follows an exponential decay of characteristic timescale $\lambda ^{-1}$, i.e. $\rho (t)=\exp {(-\lambda t)}$, yielding a red-noise type Fourier spectrum $F(\omega )\propto 1/(\omega ^2+\lambda ^2)$, a form rather similar to the spectra in Fig. 6 (up to threshold frequency $f^*$). From (6) one can also derive the associated Fokker–Planck equations for the evolution of the PDF of *x*, and the moments of the distribution can be obtained. Sura^22^ showed that—unlike in a normal distribution—the skewness and kurtosis of the PDF are non-zero and fulfil the following nontrivial general relation: $K\ge (3/2) S^2$. This holds for the values of *K* and *S* for all of our experiments, as demonstrated by the fact that all data points in Fig. 3 scatter above the dashed parabola marking $K = (3/2) S^2$.

Our analysis of the timescale associated with the largest persistence (Fig. 7), as well as the aforementioned observation of the shifting cut-off frequencies in the spectra (Fig. 6) both point to the fact that with increasing $\langle \Delta T \rangle$ a wider spectral domain is characterized by red-noise-like behavior (instead of being uncorrelated white noise). In agreement with the qualitative predictions of the CAM model and also with our observations, this spectral change is accompanied with the widening of the PDFs which follow non-normal distributions with heavy tails.

The observed $\langle \Delta T \rangle$ and $\Omega$-dependence of the largest “jumps” in the temperature time series (Fig. 5) teaches us another lesson. With the dynamic scaling arguments applied there we concluded that the distribution of the observed temperature changes in the pointwise temperature records ($\sim \partial T/\partial t$) are dominantly attributed to advection, or “hydrodynamic” processes related to the circulation ($\sim \vec {u}\nabla T$), whereas “thermodynamics”, i.e. direct conductive heat exchange between given water parcels and their vicinity ($\sim dT/dt$) plays a much lesser role on the timescales relevant for the “weather” in the tank. This finding may have relevance for the better understanding of the actual climate system, where it is still unclear whether the character of future heatwaves and extreme hot periods will have more to do with direct thermodynamic effects (due to global warming) or may rather be driven by the reorganizing atmospheric circulation. This question can be answered only with a thermodynamic theory of the mean stratification and meridional temperature gradient of the atmosphere. The dynamic part, namely the eddy adjustment to the environmental state, will then ultimately determine the frequency of heat waves. However, a theory for these processes is, to our knowledge, still lacking^33^. It is to be emphasized that laboratory experiments, like the ones of the present work are obviously not suitable to model the complex thermodynamics of atmospheric circulation where the water cycle and the associated latent heat exchange between the different layers of the troposphere (and between the troposphere and the stratosphere) contribute substantially to setting the aforementioned meridional temperature contrast and stratification. Heat waves and cold outbreaks are often associated with Rossby waves, and it is also frequently stated in the literature^1–5^ that polar amplification affects the weather at mid-latitudes markedly. The results from minimalistic experiments like ours may be of importance for the climate science community, since we can isolate weather variability related to Rossby waves. Thus, these findings may help disentangle some of the complex causal links in the climate system by providing a “test case” in which temperature fluctuations arise solely due to the flows governed by the baroclinic instability of the model atmosphere, and where the climate forcing—represented here by the average temperature contrast $\langle \Delta T \rangle$—can be considered stationary.

An earlier work by our team already investigated a similar minimal model of the climate system through an ensemble of experiments in which the thermal forcing $\Delta T (t)$ was strongly time-dependent but identical throughout a series of runs^18^. That campaign demonstrated the importance of an ensemble approach in understanding climate-like dynamics. For highly nonlinear systems subject to time-dependent control parameter drift, long-time statistics of a single “realization” cannot be considered to be appropriate surrogates of a truly meaningful probability distribution over an ensemble of “parallel realizations”^34–37^. To better understand the findings reported by Vincze et al. (2017)^18^, it was necessary to conduct the long-run experiments discussed in the present work with different settings of stationary forcing, to provide statistics with which the earlier time-dependent forcing scenarios can be compared. The new results underline the conclusion of the earlier paper that, although the “ensemble standard deviation” of the observed temperature fluctuations—i.e. the spread of the possible outcomes with decreasing $\Delta T (t)$—increases, this does not imply that the range of fluctuations within a single realization would increase. On the contrary, the Rossby wave related temperature fluctuation intervals would decrease for a smaller $\langle \Delta T \rangle$, a result that may be of relevance for better understanding the ongoing climatic processes related to Arctic amplification.

However, it is to be emphasized that our experiments did not intend to represent the global atmospheric circulation in its vast complexity with the plethora of phenomena that affect the actual local temperature records. Yet, as shown by previous research, the rotating annulus configuration provides an accurate insight into certain basic statistical properties of atmospheric temperature fluctuations^12^, and even their extreme statistics^19,20^.

## Data Availability

Measured raw data are available upon request from the corresponding author (M. Vincze).
